# The History and Capabilities of Herbal Simples

**Published:** 1891-03-28

**Authors:** W. T. Fernie


### The History and Capabilities of Herbal Simples.

By W. T. Fernie, M.D.

### XXIX.—DILL.

"What have you for dinner, good Mrs. Bond?" "Ducks in the larder and geese in the pond." So said this worthy dame in the time of our childhood; and she straightway went to the water-side calling "Dilly! dilly! come and be killed." Her careful thoughts were probably intent not only on the doomed birds, but likewise on herbs in her garden, including the spicy aromatic Dill, wherewith choice stuffing might be skilfully made to prevent indigestion with her guests from the rich repast she provided. Because cordial waters, distilled from the fragrant Dill—Anethum graveolens—are, as every mother and monthly nurse well knows, a sovereign remedy against wind in the infant, whilst equally admirable for subduing flatulence in the grown-up gourmet. The highly scented herb is an annual plant, of Asiatic origin, which grows wild in some parts of England, and is commonly cultivated in our gardens for kitchen or medicinal uses. "It hath a little stalk of a cubit high, round, and joyned, whereupon do grow leaves very finely cut, like to those of fennel, but much smaller." Its name, Dill, is derived fron the Saxon verb "dilla," to lull, because of its tranquillising properties, and its causing children to sleep. Dioscorides gave the oil from the flowers for rheumatic pains and sciatica, also a carminative water distilled from the fruit for increasing the milk of wet nurses, and for appeasing the windy belly-aches of babies. The plant is of the Umbelliferous order, and its fruit chemically furnishes Anethol, a volatile empyreumatic oil similar to that contained in the anise and caraway. Virgil in his second Eclogue speaks of the dill as the "bene olentis anethi," a pleasant and fragrant herb. Its seeds were formerly directed to be used by the pharmacopeias of London and Edinburgh. Forestus extols them for allaying sickness and hiccough. Gerarde says, Dill stayeth the yeox, or hicquet, as Dioscorides hath taught. Moreover, a teaspoonful of the bruised seeds, if boiled in water and taken hot, with bread soaked therein, wonderfully helps such as are languishing from hardened excrements, even though they may have vomited up their fæces. The name An ethum was a radical term applied by the Greeks to the plant; and it is still called Anet in some of our country districts. The pungent essential oil obtained from it consists of a hydrocarbon carvene, together with an oxygenated oil. It is a "gallant expeller of the wind, and provoker of the terms." Limbs that are swollen and cold, if rubbed with the oil of dill, are much eased, if not cured thereby. A dose of the essential oil, if given for flatulent indigestion, should be from two to five drops, on sugar or in milk. Of the distilled water sweetened, one or two teaspoonfuls may be given to an infant. The plant is largely grown in the East Indies, where it is known as Soyah. Its fruit is used there for flavouring pickles, and its water is given to parturient women. Drayton speaks of the Dill as a magic ingredient in love potions. Also in Sir Walter Scott's "Guy Mannering" the weird gipsy, Meg Merrilies, croons a cradle song at the birth of Harry Bertram, in which she says:—

"Trefoil, Vervain, John's Wort, Dill,
Hinder witches of their will."

